# Patterns of oral squamous cell carcinoma among smokeless tobacco users: A clinical study

**DOI:** 10.6026/973206300213314

**Published:** 2025-09-30

**Authors:** Damarasingu Rajesh, Meghana Voddiraju, Anil Managutti, Venkat Hemant Akurati, Jagadish Prasad Rajguru, Ancy Kuriakose, Heena Dixit, Rahul Tiwari

**Affiliations:** 1 Department of Oral and Maxillofacial Surgery, Narsinhbhai Patel Dental College and Hospital, Sankalchand Patel University, Visnagar, Gujarat, India; 2 Department of General Medicine, Government Medical College, Nizamabad, Telangana, India; 3 General Dentistry, University of Louisville, School of Dentistry, Louisville, Kentucky, USA; 4 Department of Oral and Maxillofacial Pathology, Hi-Tech Dental College and Hospital, Bhubaneswar, Odisha, India; 5 Department of oral medicine and radiology, St. Gregorios dental college, Kothamangalam, Chelad, Kerala, India; 6 Department of Medical Health Administration, Index Institute, Malwanchal University, Index City, Nemawar Road, Indore, Madhya Pradesh, India

**Keywords:** Oral squamous cell carcinoma, smokeless tobacco, gingivobuccal complex, mandibular neoplasms, survival analysis

## Abstract

Subsite distribution, mandibular invasion patterns, nodal involvement and survival outcomes in oral squamous cell carcinoma (OSCC) patients with a history of smokeless tobacco (SLT) use is of interest. Gingivobuccal complex (GBC) subsites were predominant, with frequent mandibular marrow involvement. Two- and five-year overall survival rates approximated 95% and 79%, respectively. Thus, data shows the clinical behavior of SLT-associated OSCC and underscore the importance of targeted imaging and early detection.

## Background:

Oral squamous cell carcinoma (OSCC) is frequently linked to habitual placement of smokeless tobacco products, resulting in a distinct clinical pattern that differs from smoking-related disease [1]. In regions with high SLT use, the gingivobuccal complex (GBC), including buccal mucosa and lower alveolus, is the most commonly affected subsite [5]. Retrospective clinical studies have shown that SLT-associated OSCC in the GBC tends to present with early mandibular invasion, often involving marrow or the mandibular canal, which may not be adequately predicted by standard imaging modalities [3, 4]. Recent audits of GBC OSCC cohorts treated surgically report two-year and five-year survival rates exceeding 90% and 75%, respectively, with factors like nodal metastasis and perineural invasion significantly impacting prognosis [2, 1]. Overall, SLT-related OSCC exhibits a consistent topographic and prognostic profile that necessitates precise clinical assessment [6, 7]. Therefore, it is of interest to study of oral squamous cell carcinoma in smokeless tobacco users.

## Materials and Methods:

This clinical observational study analyzed medical records of OSCC patients with documented history of smokeless tobacco use. Cases were selected based on histopathologic confirmation of primary OSCC in the oral cavity and self-reported habitual SLT consumption. Data extracted included tumour subsite, mandibular bone involvement (cortical vs marrow/canal), cervical nodal status, treatment modality (surgical with or without adjuvant therapy) and survival outcomes (overall survival at two and five years). Imaging features were reviewed to correlate preoperative radiologic invasion with histopathologic findings. Patients were followed until death or last contact and survival was estimated using Kaplan-Meier methods. Ethical clearance was obtained and confidentiality measures were maintained.

## Results:

A total of 120 SLT-associated OSCC cases were studied. The median age was 58 years (range 40-75), with 72% males. The analysis of subsite distribution revealed that the gingivobuccal complex (GBC) was the most frequently affected region, accounting for nearly two-thirds (65%) of smokeless tobacco-associated oral squamous cell carcinoma (OSCC) cases. The tongue and floor of the mouth together represented 21% of cases, emerging as the second most common site of involvement. Other subsites of the oral cavity were comparatively less affected, comprising only 14% of the study population (Table 1 - see PDF). Evaluation of nodal involvement and survival outcomes showed that clinically positive neck nodes were more common in patients with GBC lesions (59%) compared to those with non-GBC sites (43%). Despite this higher nodal burden, survival outcomes were more favorable in the GBC group, with a two-year overall survival rate of 95% compared to 83% in the non-GBC group. Long-term outcomes also demonstrated a survival advantage for GBC cases, with a five-year overall survival of 78% versus only 57% among patients with non-GBC lesions (Table 2 - see PDF).

## Discussion:

The survival data, with two- and five-year overall survival rates of approximately 95% and 79%, align with recent surgical series of GBC OSCC cohorts, which reported similar outcomes and identified nodal metastasis and perineural invasion as key prognostic indicators [8, 9]. Patterns of mandibular invasion in the GBC, especially marrow and canal involvement, are predictive of poorer outcomes and support the needed for comprehensive bone-margin planning [10]. Imaging modalities, including high-resolution CT and MRI, have variable accuracy in detecting marrow invasion, leading to underestimation of disease extent in up to 15% of cases, consistent with the literature [11]. The higher incidence of nodal metastasis observed in GBC versus non-GBC sites reinforces anatomical drainage patterns unique to SLT-associated lesions and the necessity for elective neck dissection in lesions thicker than 4 mm or clinically node-negative but high-risk by subsite [12]. Early detection through visual screening and public awareness, particularly in high-SLT populations, remains vital given that advanced stage at presentation is associated with significantly worse survival, often dipping to 20%-30% in such cohorts [13]. Recent registry-based analyses indicate that oral cavity cancers in SLT-endemic regions often present at advanced stages, contributing to variable five-year survival rates across institutions, ranging from 55% to 80%, depending on access to timely surgery and adjuvant therapy [14]. This underscores disparities in health system responsiveness and the role of delays in undermining outcomes. Emerging concepts such as the Marrow and Mandibular Canal (MMC) staging system, which stratifies mandibular invasion extent more precisely than conventional T-staging, have shown promise in improving prognostication and guiding surgical margin decisions [15]. Integration of MMC concepts into standard practice could refine surgical approaches and potentially enhance local control without overtreatment. In sum, SLT-associated OSCC exhibits a distinct clinical pattern-predominantly affecting the gingivobuccal complex, exhibiting frequent marrow/canal invasion and bearing a high nodal burden. While survival can be favorable with appropriate intervention, optimising imaging, embracing MMC staging frameworks and facilitating early detection are critical to improving outcomes across diverse clinical settings.

## Conclusion:

SLT-associated OSCC demonstrates a consistent clinico-pathologic profile: gingivobuccal predilection, frequent mandibular marrow involvement, substantial nodal disease and overall favorable survival with timely surgical management. Enhanced imaging, MMC-based planning and early detection strategies are key to optimizing patient outcomes.
